# DiD IT?: a differences-in-differences investigation tool to quantify the impact of local incidents on public health using real-time syndromic surveillance health data

**DOI:** 10.1017/S0950268823000444

**Published:** 2023-03-15

**Authors:** Roger Morbey, Dan Todkill, Daniella DeAngelis, Andre Charlett, Alex Elliot

**Affiliations:** 1 Real-time Syndromic Surveillance Team, Field Services, Health Protection Operations, UK Health Security Agency, Birmingham, UK; 2 UKHSA, Statistics, Modelling & Economics, Data Analytics & Surveillance, London, UK

**Keywords:** Causal inference, epidemiology, outbreak detection

## Abstract

Syndromic surveillance was originally developed to provide early warning compared to laboratory surveillance, but it is increasing used for real-time situational awareness. When a potential threat to public health is identified, a rapid assessment of its impact is required for public health management. When threats are localised, analysis is more complex as local trends need to be separated from national trends and differences compared to unaffected areas may be due to confounding factors such as deprivation or age distributions. Accounting for confounding factors usually requires an in-depth study, which takes time. Therefore, a tool is required which can provide a rapid estimate of local incidents using syndromic surveillance data.

Here, we present ‘DiD IT?’, a new investigation tool designed to measure the impact of local threats to public health. ‘DiD IT?’ uses a difference-in-differences statistical approach to account for temporal and spatial confounding and provide a direct estimate of impact due to incidents. Temporal confounding differences are estimated by comparing unaffected locations during and outside of exposure periods. Whilst spatial confounding differences are estimated by comparing unaffected and exposed locations outside of the exposure period. Any remaining differences can be considered to be the direct effect of the local incident.

We illustrate the potential utility of the tool through four examples of localised health protection incidents in England. The examples cover a range of data sources including general practitioner (GP) consultations, emergency department (ED) attendances and a telehealth call and online health symptom checker; and different types of incidents including, infectious disease outbreak, mass-gathering, extreme weather and an industrial fire. The examples use the UK Health Security Agency's ongoing real-time syndromic surveillance systems to show how results can be obtained in near real-time.

The tool identified 700 additional online difficulty breathing assessments associated with a severe thunderstorm, 53 additional GP consultations during a mumps outbreak, 2–3 telehealth line calls following an industrial fire and that there was no significant increase in ED attendances during the G7 summit in 2021.

DiD IT? can provide estimates for the direct impact of localised events in real-time as part of a syndromic surveillance system. Thus, it has the potential for enhancing surveillance and can be used to evaluate the effectiveness of extending national surveillance to a more granular local surveillance.

## Background

Public health surveillance involves monitoring a wide range of data sources (both health and non-health) to identify where there has been an adverse impact on the population's health. When a potential threat or increased risk is identified, a rapid assessment may be needed to assess the scale of any impact on the population's health. Syndromic surveillance involves monitoring diagnoses or symptoms in conjunction with traditional surveillance, e.g. laboratory surveillance or field epidemiology [1, 2]. Whilst the originating objective for syndromic surveillance was to provide early warning of seasonal outbreaks, it has increasingly been found to be useful for real-time situational awareness. Therefore, syndromic surveillance systems require, not just outbreak detection methods but tools that can quantify the impacts of events in real-time.

Syndromic surveillance has primarily focussed on events that have the largest public health impacts, like seasonal influenza. Thus, surveillance has been at a national level, with regional data used to monitor geographical variations. However, there is increasing interest in sub-national outbreaks and events [3–5]. Thomas *et al*., evaluated the utility of local syndromic surveillance systems and found that syndromic surveillance was being used for situational awareness of local impacts rather than for routine localised outbreak detection [6]. Syndromic surveillance has proven to be useful during nationwide epidemics and mass-gatherings, but it is less clear how effective it is at assessing local impacts where the numbers involved are much smaller [7, 8]. Consequently, public reporting tends to focus on national and regional surveillance, e.g. https://www.gov.uk/government/collections/syndromic-surveillance-systems-and-analyses.

UKHSA coordinates six national syndromic surveillance data sources to monitor potential threats to public health in real-time. Calls to a national telephone helpline (NHS 111) [9] and an online NHS 111 symptom checker are reviewed daily, along with general practitioner (GP) consultations from both scheduled in-hours and unscheduled out-of-hours [10] activity. Also, data are collected on ambulance dispatch calls [11] and emergency department (ED) attendances [12]. Daily surveillance involves reviewing key syndrome national and regional trends [13] as well as an automated statistical outbreak detection algorithm [14]. Whilst routine monitoring of syndromic data focuses on potential national threats, there is also the ability to investigate local data when requested by a local public health official concerned about a potential local threat.

A key issue when investigating local syndromic data is whether changes can be attributed to a specific cause. For example, are increasing trends in respiratory symptoms seen during a major sporting event due to an associated increase in social mixing or normal seasonal variation? Similarly, are rates of eye problems above national average levels near a wildfire due to the fire or due to other factors specific to the local community? Accounting for confounding spatial or temporal factors that may bias results requires careful research that takes time and thus cannot produce the rapid assessments required by decision makers. Therefore, as part of the national programme of real-time syndromic surveillance in the UK Health Security Agency (UKHSA) we have developed a novel investigation tool to provide a rapid estimate for the direct effect of a localised public health threat. We use a causal inference ‘difference-in-differences’ (DiD) [15] approach which accounts for spatial and temporal differences without the necessity of identifying the impact of individual confounding factors like deprivation or rurality.

The DiD method involves comparing cases and controls across two dimensions, in our case time and space. Therefore, we collected data not just from locations ‘exposed’ to the potential health hazard but also other control locations that are outside the exposure zone. Similarly, data was not just collected during the period of exposure but also from a ‘control’ period, usually immediately prior to the start of the exposure. It is preferable that the control period is as close as possible to the exposure period so that the data is less likely to be biased by any long-term trends. A period prior rather than after the exposure is operationally preferable because the tool may be used before the potential threat has ended.

We illustrate the wide potential application of the new DiD Investigation Tool (DiD IT?) through four examples of incidents that occurred in England: a local mumps outbreak in students within the university city of Nottingham [16]; the 2021 G7 summit in Cornwall; a thunderstorm asthma event in the South East [17]; and a large industrial fire.

## Methods

### Statistical analysis

All the case studies detailed below were in the form of daily counts of syndromic indicators aggregated to local areas. Variables were created for days and location, along with binary variables to indicate the exposure period and which locations were within the area of each incident.

We used a Negative Binomial Regression model as the syndromic data are counts in a health setting, and this model is robust for both over-dispersion and the possibility of a high proportion of zero counts in local data. The regression model was in form as shown in equation 1:1
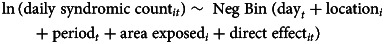


The subscript i refers to all the different locations and t to the days studied. The variable day is a separate factor for each day in the study and similarly location is a factor for each location. The binary variable ‘period’ is a 1 during the exposure period, 0 otherwise. Similarly, the ‘area exposed’ variable is a 1 for locations that were exposed, 0 otherwise. The ‘direct effect’ variable is the product of the ‘period’ and ‘area exposed’ binary variables.

The regression coefficients for the binary variables, ‘period’, ‘area exposed’ and ‘direct effect’ are estimates respectively for:

- The difference between the exposure period and the control period that is not due to the incident and is the same for all locations, exposed and unexposed
- The difference between the exposed and unexposed locations that is unaffected by the incident and the same during and outside of the exposure period
- The direct effect of the incident, having taken into account any potential confounding factors due to temporal or spatial differences

The direct effect coefficient gives an estimate for the rate ratio, comparing rates in exposed areas during events to a counterfactual as if the event had not occurred. Also, the number of additional counts presenting to a syndromic system due to the event can be estimated. 95% statistically significant confidence intervals were calculated around the direct effect, using boot strap methods whenever direct computation was not possible.

### Case studies

#### Example 1: local mumps outbreak

On 26th February 2019, syndromic surveillance systems prospectively detected an increase in GP consultations for mumps in the university city of Nottingham, England, which has a large student population. Routine prospective syndromic surveillance was able to quantify the spike in cases, however public health practitioners also require context, ideally an estimate for how many additional cases were occurring in affected areas due to the outbreak. Using DiD IT tool we compared the number of GP consultations for mumps over the following 17 days with the previous 17-day period, comparing Nottingham upper tier local authority (UTLA) with 73 other UTLAs where at least one mumps consultations had been recorded.

#### Example 2: G7 economic summit in Cornwall

On 11–13th June 2021 the G7 summit was held in Carbis Bay, Cornwall, England. The G7 summit presented a significant mass gathering and real-time syndromic surveillance provided support to an enhanced surveillance programme supporting the G7 Summit. Specifically, public health incident directors required reassurance that no significant increase in health-seeking activity was occurring during the event that might suggest a threat to public health. We compared the number of ED attendances at the two hospitals located nearest to the G7 summit venue with 178 other EDs across England, comparing the G7 summit period and subsequent seven days with a control period of the preceding ten days.

#### Example 3: outbreak of ‘thunderstorm asthma’ across South East England

On 17th June 2021 real-time syndromic surveillance systems detected large spikes in asthma and difficulty breathing indicators across a range of national syndromic surveillance systems. This coincided with several localised, weak, or moderate thunderstorms specifically across parts of South East England on the night of June 16. ‘Thunderstorm asthma’ has been observed previously in the UK and other countries in the World, whereby certain weather and environmental conditions can trigger a surge in patients presenting with exacerbated symptoms of asthma [18]. We need to quantify the health impact of thunderstorm asthma to support research into the impact of such events and allow public health practitioners to provide alerts ahead of thunderstorm asthma episodes. We defined the exposure period as Thursday 17th June 2021 and control dates as the Thursdays immediately before and afterwards (10th and 24th June). The exposure location was taken as all London boroughs and contiguous upper tier local authorities neighbouring the s.e. coast, including Cambridgeshire, Essex, Hertfordshire, Suffolk, Thurrock, Southend-on-Sea, Brighton and Hove, East Sussex, Hampshire, Kent, Medway, Surrey, and West Sussex. For this study, we considered the impact on the NHS 111 online symptom checker.

#### Example 4: local impact of an industrial fire

Due to the small exposure locations and count numbers involved in this case study we have anonymised the location and time of this incident. This was a large industrial fire in an urban setting in England. During environmental incidents like this, public health officials use syndromic surveillance to provide situational awareness, to quantify whether there have been any increases in local health seeking behaviour potentially linked to the incident. We have considered the first three days after the fire started as the exposure period, compared with the previous seven days as a control. The fire occurred on the border between three postcode districts, so these three were taken as the exposure location. 1860 other English postcode districts were taken as controls. GP out-of-hours and unscheduled calls for difficulty breathing were used to measure the impact on public health.

## Results

The DiD IT method was able to provide estimates in each example for the risk ratio of ‘exposure’ to the potential public health threat. Confidences intervals were calculated directly where possible for each of these risk ratios, and via bootstrapping methods from the R package ‘boot’ [19] when direct methods would not converge due to small numbers in example 4.

DiD IT estimated that the mumps outbreak in Nottingham resulted in a 11.5-fold increase in the risk of patients presenting with symptoms to a GP. Between 26th Feb and 14th March 2019 we estimate that the mumps outbreak resulted in an additional 53 patients consulting GP practices in Nottingham covered by the GP syndromic surveillance system.

During the G7 summit there was a slight rise in the number of ED attendances at the two hospitals located nearest to the G7 venue, but this was not statistically significant (risk ratio of 1.05 (0.95, 1.16)). Across the ten days of the exposure period, this central estimate would be equivalent to seeing an extra 208 patients however the 95% confidence interval includes a possible decrease in the number of attendances (−191.4, 682.2).

On the 17th of June 2021 patients living in areas of the South East of England (including London) were 4.9 times more likely to use the NHS 111 app to check their symptoms of difficulty breathing. We estimate that on 17 June an additional 700 people used the app with difficulty breathing symptoms that were potentially linked to the occurrence of an episode of ‘thunderstorm asthma’.

When considering the industrial fire, we looked at NHS 111 calls for difficulty breathing at the postcode district level. At this, very localised level, on most days there were less than three difficulty breathing calls reported to NHS 111 in the exposed district. We did detect a small increase, risk ratio of 1.78 in the areas around the fire. However, this was just equivalent to between 2 and 3 additional calls in total (Table 1).

**Table 1.** Risk ratios and estimated number of additional counts due to exposure

Potential public health threat	Syndromic system/indicator	Risk ratio of direct effect	95% confidence interval	Estimated number of additional healthcare counts
1. Mumps outbreak	GP in-hours consultations for mumps	11.5	(4.08, 48.28)	53
2. G7 summit	ED attendances	1.05	(0.95, 1.16)	Not significant
3. Thunderstorm asthma	NHS 111 online assessments for difficulty breathing	4.86	(4.05, 5.83)	700.3
4. Industrial fire	NHS 111 calls for difficulty breathing	1.78	(1.13, 6.35)	2.6

**Fig. 1.**
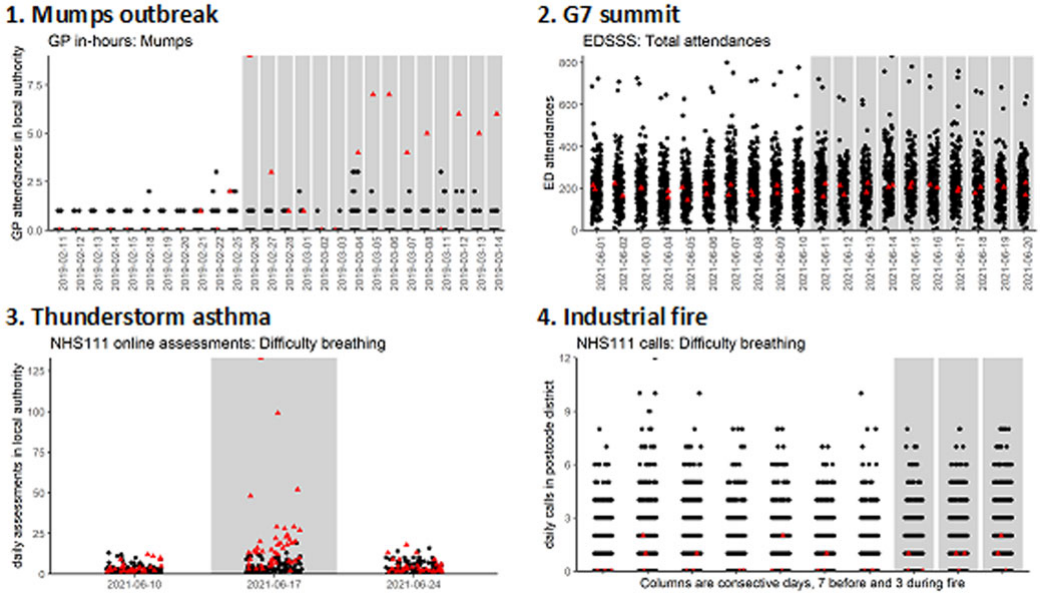
Daily counts for syndromic data around incidents, examples 1–4. Red triangles are exposed locations, black dots control locations. Grey columns show exposure periods.

Figure 1 shows the daily counts for each example, with the exposure period and exposure locations identified.

## Discussion

### Key findings

Using syndromic surveillance data to support local public health incidents can be difficult because of low numbers and temporal and spatial heterogeneity. Here we illustrate the use of a DID methodology to further enhance the potential use of syndromic data for local incident support. The thunderstorm asthma and local mumps outbreak both resulted in significantly increased risk of assessing health services and significant numbers of extra consultations. By contrast, the industrial fire, although showing a statistically significant increase, resulted in just 2–3 extra calls to a national telephone helpline over three days which is unlikely to be considered clinically significant and warrant any further public health action. Finally, the G7 summit did not result in a statistically significant increase in ED attendances. However, the context shows that if a 5% increase in ED attendances had occurred it would mean over two hundred extra patients over a ten-day period.

### Implications for public health

We have shown that using a novel DiD methodology can improve the utility of syndromic surveillance for detecting localised increases in activity. We were able to provide estimates for the direct effects of the health hazards studied. Traditional hypotheses testing or aberration detection methods can say whether an increase is unlikely to be just chance but do not answer the counterfactual question, how many additional cases were attributable to a health threat. DiD IT can provide both a risk ratio and context in terms of the number of extra cases arising from the health threat.

The management of an incident and the interventions available will vary depending on the type of incident. In this study we have therefore considered examples across a wide range of potential health threats, from infectious diseases to environmental hazards and mass gatherings. In some situations, e.g. a mass gathering, there is an increased public health risk and real-time reassurance may be required that the gathering has not resulted in any increase in health care presentation. Similarly, a local environmental hazard, like an industrial fire, may cause public concern and a real-time assessment is needed of whether there has been any increases in local people presenting with related symptoms. Alternatively, there may be an incident where there are known confirmed cases, but situational awareness is still needed to assess the wider community impact or pressures on local health care services.

DiD IT is computationally quick to apply and does not require lengthy calculations of confounding factors such as long-term demographic trends, or local differences due to deprivation etc. Importantly, DiD IT can be used operationally in real-time, even before a potential threat has ended, for instance during a mass gathering. Thus, DiD IT can become part of the routine reporting to incident directors and response teams.

### Comparison to other studies

Many existing studies of applying syndromic surveillance to local outbreaks focus on early detection, [20–22]. Whilst Thomas *et al*. found that syndromic surveillance was being used for situational awareness of local impacts rather than for routine localised outbreak detection [6]. Consequently, in addition to tools for localised outbreak detection, there is a need for tools like DiD IT which can estimate local health burden.

One of the perceived benefits of syndromic surveillance compared to traditional laboratory surveillance is that it can capture the impact of a wider population, including those with less severe outcomes. Anderson *et al*., used a range of different data sources to analyse waterborne and foodborne outbreaks in Sweden, concluding that the telephone triage data suggested the outbreak was ten times larger than officially reported [4].

A relatively under-researched area is the potential for using syndromic surveillance to measure the impact of localised interventions on demand for health care services. For example, Chalder *et al*. assess the impact of a change in local service provision [23]. The DiD IT method could potentially provide a rapid analysis of the impact of localised changes to service provision.

### Limitations

DiD IT is designed to investigate the impact of localised threats to public health. Due to the need for controls, it is not the correct tool to investigate larger incidents, which have a wider geographical impact, such as seasonal influenza. Similarly, a control period is needed and therefore DiD IT would not be suitable to measure the impact of a ‘slow burn’ event i.e. one where a gradual change in trends is observed, or an impact that took effect over many months.

Importantly, syndromic surveillance can never prove a causal link between exposure to a potential public health threat and an individual's health outcomes and subsequent healthcare seeking behaviour. Whilst DiD IT can provide an estimate for the direct effect of a cause, epidemiologists will always have to use other sources of intelligence to decide if the link is plausible e.g. through laboratory reports. Thus, DiD IT should only be used when there is an epidemiologically based rationale to believe that a potential threat could result in an increase in a related syndromic indicator. Public health practitioners should avoid the temptation to apply DiD IT to any cluster of increased counts where there is no independently verifiable cause. DiD IT is not designed to be an early warning tool, but a situational awareness support tool. By comparison, cluster analysis or scan statistics can be used for aberration detection of previously unknown local incidents. However, cluster analysis is considerably more computational complex than DiD IT and usually requires precise information about the distance between individual cases, which is not available with aggregated anonymised data. Furthermore, to estimate how much of a cluster is due to an incident, prior estimates are required for the expected number of cases in each location.

Furthermore, when using DiD IT, public health practitioners should consider whether there are any alternative threats operating in the same location at the same time. The accuracy of DiD IT will be less if there are other threats that overlap both spatially and temporally with whatever is being studied. However, it may be possible to improve the accuracy of estimates by excluding from the study locations or dates that are suspected to be affected by other threats. Similarly, if it is unclear when an exposure started or how widespread the effects are then the ‘grey area’ can be excluded. For example, only the immediate surroundings of a fire could be used for the exposure location and control locations limited to all areas that are more than, say 100 miles away.

The ability of syndromic surveillance to detect local impacts will always be dependent on the population coverage of each system and it is important not to provide a false reassurance that ‘nothing is happening’ where surveillance system coverage is poor. Also, the ability to assess the impact on public health will depend on the range of data sources available. For example, here we considered the impact of thunderstorm asthma on users of an online assessment tool, however we know there was also an impact on GP out-of-hours consultations, ambulance calls and ED attendances [17]. Assessing the full impact on public health requires a range of syndromic surveillance systems and cannot include health care providers for whom no data is available.

### Recommendations

We propose that DiD IT continues to be part of the routine syndromic surveillance analysis for public health in England and we further explore its application in syndromic surveillance and to other surveillance data. Furthermore, the tool can be used retrospectively to evaluate the local detection capability of systems against historical examples. Work is underway to create an application for use in real-time by incident directors in England and an R package could be created for use by other countries utilising syndromic surveillance as part of a public health programme.

Future planned work includes a validation of DiD IT using a combination of recorded outbreaks and simulations using synthetic ‘injects’ into historical data. The simulations will enable us to test how accurate DiD IT is at estimating the size of local incidents. Furthermore, the validation work will include sensitivity analysis to determine how selection of the control period, control locations, including any ‘washout period’ affect performance, which will feed into further iterations of the tool.

## Data Availability

The data that support the findings of this study are available from NHS Digital, and TPP. Data are available from the authors with the permission of NHS Digital and TPP.
